# CNN-Based Classification for Highly Similar Vehicle Model Using Multi-Task Learning

**DOI:** 10.3390/jimaging8110293

**Published:** 2022-10-22

**Authors:** Donny Avianto, Agus Harjoko, Afiahayati

**Affiliations:** 1 Department of Informatics, Universitas Teknologi Yogyakarta, Yogyakarta 55285, Indonesia; 2 Department of Computer Science and Electronics, Universitas Gadjah Mada, Yogyakarta 55281, Indonesia

**Keywords:** convolutional neural network, vehicle make and model, multi-task learning

## Abstract

Vehicle make and model classification is crucial to the operation of an intelligent transportation system (ITS). Fine-grained vehicle information such as make and model can help officers uncover cases of traffic violations when license plate information cannot be obtained. Various techniques have been developed to perform vehicle make and model classification. However, it is very hard to identify the make and model of vehicles with highly similar visual appearances. The classifier contains a lot of potential for mistakes because the vehicles look very similar but have different models and manufacturers. To solve this problem, a fine-grained classifier based on convolutional neural networks with a multi-task learning approach is proposed in this paper. The proposed method takes a vehicle image as input and extracts features using the VGG-16 architecture. The extracted features will then be sent to two different branches, with one branch being used to classify the vehicle model and the other to classify the vehicle make. The performance of the proposed method was evaluated using the InaV-Dash dataset, which contains an Indonesian vehicle model with a highly similar visual appearance. The experimental results show that the proposed method achieves 98.73% accuracy for vehicle make and 97.69% accuracy for vehicle model. Our study also demonstrates that the proposed method is able to improve the performance of the baseline method on highly similar vehicle classification problems.

## 1. Introduction

The advent of Artificial Intelligence (AI) has significantly altered our world. AI reaches numerous sectors of our lives, including medicine [1], education [2], mining [3], and transportation [4]. The intelligent transportation system (ITS) is an example of how AI is used in the transportation sector. The ability of an ITS to read license plates can help authorized officers with vehicle surveillance and traffic law enforcement. However, license plate information can be difficult or impossible to obtain under some conditions, such as: not all roads being equipped with surveillance cameras; the physical condition of the license plates being badly damaged; or the use of fake license plates on vehicles. Besides the license plate, the vehicle make and vehicle model are also important pieces of vehicle information [5]. 

Vehicle make and vehicle model information can be acquired more easily based on their physical appearances. In addition, it is still possible to acquire this information even if the vehicle is at a significant distance. A module that can recognize the vehicle’s make and model should help officers find the vehicle more rapidly. Researchers have come up with many different methods to identify the vehicle make and model, such as using K-nearest neighbors [6], 3D models [7], and deep learning [8,9,10,11]. Despite the fact that numerous methods have been presented in the past, this problem remains unique due to factors such as the traffic flow, the state of the transportation infrastructure, and the variety of vehicles in each country. 

In a country such as Indonesia, for example, surveillance cameras are still only installed on major roads and in major cities. Additionally, the fact that the majority of vehicles on Indonesian roads are highly similar poses a further challenge. Highly similar vehicles are vehicles made by different manufacturers and that have different model names, but that are very similar in terms of physical appearance. This condition can confuse the system and lead to a high rate of misclassification. So far, only a small amount of research has been performed on the topic of identifying the make and model of highly similar vehicles.

This study proposes a method based on deep learning for vehicle make and model recognition, particularly for highly similar vehicles. This study combines vehicle manufacturer information into the learning process through a multi-task learning approach in order to address highly similar vehicles. This is possible due to the close association between manufacturer information and car model. By concurrently exploring the manufacturer and model under a supervised learning process, vehicle manufacturer data will naturally provide hints to the vehicle model and enable the network to make more accurate predictions. This study uses the InaV-Dash dataset, which was collected using a dashboard camera, as a potential solution to the limited number of surveillance cameras in Indonesia. In brief, the main contributions of this study are:

1. Modifications of the CNN architecture by adding multi-task learning have increased the efficiency of the classification because it can provide two types of output (one for vehicle make and one for vehicle model) in one recognition cycle;
2. Investigation of multiple CNN base architectures to perform multi-task learning for two tasks (vehicle brand and vehicle model classification) on highly similar vehicles has resulted in improved performance for all tasks across all CNN base architectures;
3. Evaluation of the proposed method employing a dataset of images captured by a dashboard camera has provided a new and unique perspective in comparison to other existing car datasets.

Numerous experiments have been conducted to evaluate the proposed architectures. All of the baseline architectures that were used in this research showed that the proposed network architecture is both efficient and able to improve accuracy.

The paper is organized as follows: Section 2 discusses related work on the classification of vehicle makes and models, fine-grained classification, and multi-task learning. Section 3 presents the InaV-Dash dataset’s characteristics and statistics. Section 4 discusses some of the theoretical background of the proposed architecture for highly similar vehicle classifications. Section 4 contains further information on the proposed architecture and composition of each layer. Section 5 discusses how to train and evaluate the classification system’s performance. Section 6 will bring the research to a close.

## 2. Related Works

Numerous academics have proposed methods for vehicle make and model recognition. Based on the method, vehicle make and model recognition may be divided into two categories: a hand-crafted feature approach and a deep learning feature approach. The hand-crafted feature approach utilizes a collection of descriptors to extract significant vehicle features from an image. Then, these features are given to a classifier. Some hand-crafted feature approaches that are often used for vehicle make and model recognition are: Scale Invariant Transform Feature (SIFT) [12,13], Histogram of Oriented Gradients (HOGs) [14,15,16,17], and Speeded-Up Robust Features (SURFs) [18,19].

In contrast to the hand-crafted feature approach, which relies heavily on human-engineered feature extractors, the deep learning feature approach does not need a human to create features. The deep learning feature approach will make important features directly from the data that is input through the learning process. This means that the feature that comes out of it can improve the classification performance in almost every case, including vehicle make and model recognition. The use of a deep learning approach for vehicle make and model recognition has been proposed by several researchers. They use different CNN architectures, including: ResNet50 [20,21], ResNet101 [22], VGG-16 [23,24], and Inception [25,26]. In earlier work, researchers measured performance using datasets such as Stanford Cars, CompCars, and CompCars Surveillance. The results of their studies show that deep learning features are more accurate than hand-crafted features. This also shows that CNN’s features are very good at recognizing the make and model of a car.

However, in most previous studies, vehicle make information was obtained automatically by the classifier based on the vehicle model predictions. In fact, knowing the manufacturer or vehicle make is an important part of distinguishing between highly similar vehicles, as it can lead to the correct vehicle model class. Up until now, only a few pieces of research have examined the use of dedicated networks to generate predictions about vehicle makes as an output. This is because using a dedicated network to predict the manufacturer of a vehicle is seen as having a lot of drawbacks, such as increasing the cost of computation, taking more time, and making the system more complicated. Therefore, in this study, a multi-task learning (MTL) approach will be used in the deep learning approach as a way to build vehicle make and vehicle model classifiers separately without making the system much more complicated.

MTL can be considered as one technique for machines to emulate human learning processes, as people frequently transfer information across tasks and vice versa when these tasks are connected. In terms of classification, MTL aims to improve classifier learning by combining knowledge from several tasks [27]. The use of MTL in deep learning methods has been carried out by several researchers in various fields, including vehicle classification. Huo et al. used MTL on the region-based convolutional neural network (RCNN) to recognize three tasks: the type of vehicle (sedan, van, bus, truck), the point of view (front, front-side, back-side), and the lighting conditions (day or night) [28]. The method proposed in that research is evaluated using an own-collected dataset taken using traffic surveillance cameras. The evaluation results show that the multi-task CNN model is very good at solving the problem of multi-view vehicle type classification. Xia et al. used CNN with MTL to recognize vehicle manufacturer logos [29]. The proposed system is not only capable of outputting the vehicle make as an output but also other attributes related to logo visuals such as alphabet, symmetry, encircled, and animal-like. A vehicle logo database with 780 images from 15 different vehicle manufacturers was used to measure the performance of the proposed system. The proposed multi-task CNN model works well for both logo classification and attribution prediction, with an overall accuracy of 98.14%. 

Sun et al. proposed a CNN multi-task method with a soft sharing mechanism that can recognize vehicle models and vehicle colors [30]. The soft sharing mechanism means that each task will have a different architecture. In the proposed architecture, the vehicle model recognition task uses the dense block, while the vehicle color recognition task uses the convolutional block. The system evaluation was conducted using the PKU-VD dataset, which was filtered to leave 201 vehicle models and 11 color types, for a total of 500,000 images. The evaluation results indicate that the suggested architecture is capable of simultaneously learning multiple vehicle properties, which enriches the output and enables it to achieve a high accuracy (above 94%) for all tasks. However, because each task has its own architecture, the proposed system becomes more complicated and requires a lot of computational power. In addition, this study does not go into detail about how similar the recognized vehicles are, so it does not consider vehicle make information to be an essential component of classifying vehicle models.

## 3. Dataset

### InaV-Dash Dataset

**Figure 1:**
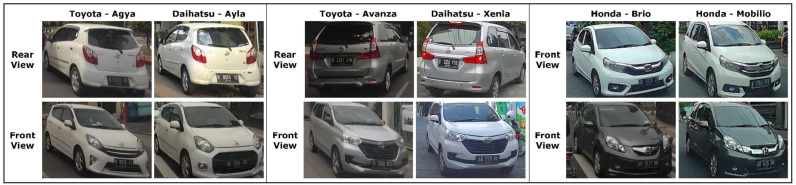
A pair of vehicle model images that are highly similar.

In this study, we collected vehicle images to form a dataset called the Indonesian Vehicle Dashboard Dataset (InaV-Dash dataset). This dataset has different characteristics from other common datasets, such as CompCars and Stanford Cars. The first characteristic to know about this dataset is that the cars that were collected for it are highly similar, as explained in the first section. This is intended to adjust to the road conditions in Indonesia, which are dominated by highly similar vehicles. Therefore, this dataset only consists of four vehicle makes and 10 vehicle models, with a total of 4192 images divided into 2934 training images and 1258 test images. Figure 1 shows some examples of pairs of cars that have a high degree of similarity.

Based on Figure 1, it can be seen that the main difference between the pair of vehicle models is in the logo emblem, which refers to the vehicle make. Due to this, information about the vehicle’s make is considered important for correctly classifying the model.

**Figure 2:**
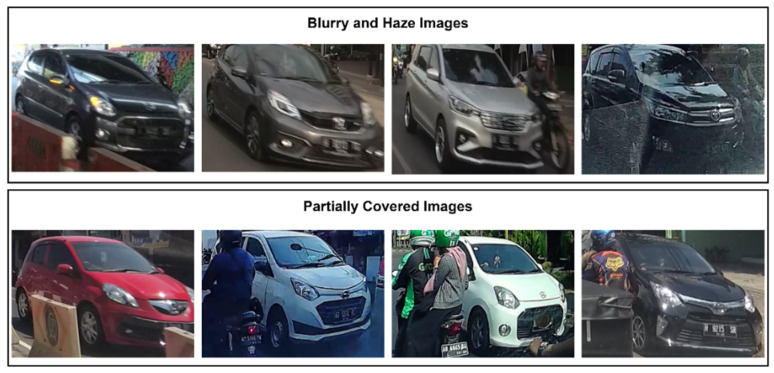
Some examples of challenging images contained in the InaV-Dash Dataset.

The second characteristic of this set of data is that it was collected using a dashboard camera in a real-life traffic scene. As depicted in Figure 2, the usage of the dashboard camera presents an additional difficulty as some vehicle images look hazy and others are obscured by other objects or cars.

**Figure 3:**
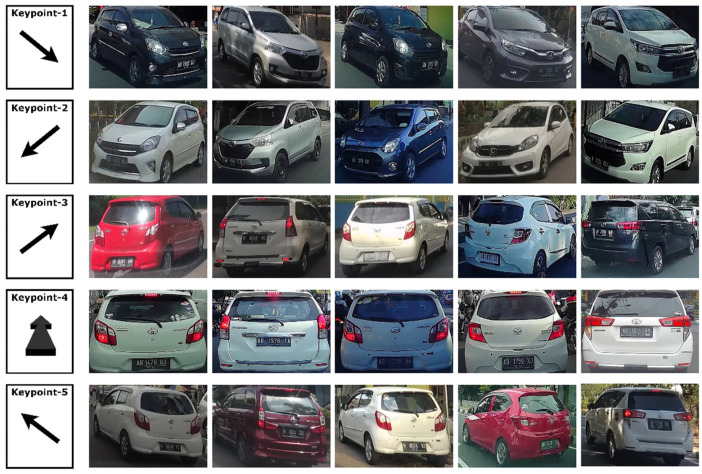
The five key points in the InaV-Dash Dataset.

The dashboard camera used is set at a Full HD resolution of 1920 × 1080 pixels and runs at 60 frames per second (fps). Full HD resolution is needed to keep the details on small parts such as logo emblems, and 60 fps is needed to keep the image from being blurry due to how fast the vehicle is moving. This is very different from the image characteristics in the CompCars and Standford Cars datasets, which are generally acquired when the vehicle is stationary or parked using an SLR camera with more complete setting options. The use of the dashboard camera also provides five possible key points for each vehicle model that will be recognized. Figure 3 shows examples of the five key points in this dataset, and Table 1 shows detailed statistics of the dataset used in this study.

## 4. Proposed Method

This section discusses the methodology used to build the proposed method, beginning with a discussion of the numerous theories utilized to rationalize the modifications. Then, all of the modifications made to the proposed method architecture, including the type of layer, the number of neurons, and the activation function, are explained in great depth in the following sections. Finally, the methods for evaluating the proposed method’s performance are explained in detail.

### 4.1. Single-Task and Multi-Task Learning

**Figure 4:**
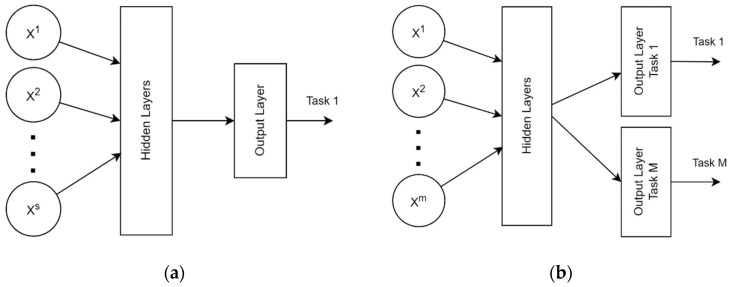
Artificial neural network architecture with (**a**) single-task learning approach; (**b**) multi-task learning approach.

In most studies, the proposed artificial neural network is trained using a single task learning (STL) method like the study conducted by [31,32]. This means that if we want to perform another classification task on STL, we then need to develop a new network with a customized output layer to learn the new task. This approach is considered to be less effective, especially if both the new and old tasks use the same input. Therefore, a new approach, namely multi-task learning (MTL), was developed to overcome this inefficiency by using the knowledge from numerous related tasks and improving the generalization performance of all the tasks [33]. This approach also has the capacity for incremental learning, which allows for the subnet of a new task to be added without updating previously learned parameters [34]. Figure 4 shows the main differences between the STL and MTL approaches in terms of network architecture. 

In this study, the MTL approach will be used to classify four vehicle brand classes and ten vehicle model classes. The use of MTL is intended to improve classification performance, especially for the vehicle model classification task in the case of highly similar vehicles. The two tasks are considered closely related because recognizing the vehicle brand can help identify the vehicle model, especially for highly similar vehicles.

### 4.2. Convolutional Neural Network

The emergence of CNN can make up for the shortcomings of conventional machine learning methods, which are only able to process data in their original raw form. According to [35], CNNs with three or more layers can learn very complex functions and provide image features needed for classification [36,37]. CNN is the most popular method for classifying images at the moment [38], including classifying vehicles [39,40,41,42], and there are many different CNN architectures for many different purposes [43]. In this study, several CNN architectures will be used, such as VGG [44], ResNet [45], Inception [46], and MobileNet.

The Visual Geometry Group (VGG) architecture is made up of multiple blocks with stacked convolution layers, a max-pooling layer, and three fully connected layers. The use of this large number of layers (deep CNN) often leads to accuracy degradation problems. The Residual Network (ResNet) is an architecture that was developed to overcome the problems of using residual learning blocks and shortcut connections [47]. Google announced CNN’s Inception architecture, which uses Inception blocks [48] and a learning strategy from [49] to solve various object size problems. Since its introduction, Inception has been updated with Inception-v2 and Inception-ResNet. Inception-v2 uses less memory and less computational load than the original [50], while Inception-ResNet can be trained faster [51,52] even though it has a deeper network. MobileNet is optimized for mobile and embedded applications, so it is compliant with resource limitations [53,54,55]. The best thing about MobileNet lies in its multipliers and depth-wise separable convolution that minimize parameters and computation [56,57].

### 4.3. Proposed Multi-Task Convolutional Neural Network

**Figure 5:**
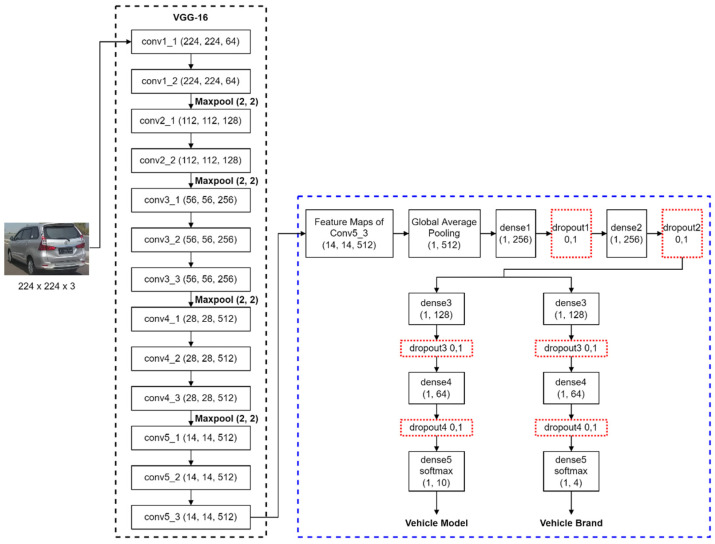
Architecture of proposed multi-task CNN.

This paper proposed a modification to the CNN architecture to make it easier to classify the make and model of highly similar vehicles. The modifications made include implementing the MTL approach to increase CNN efficiency. The proposed architecture can be seen in Figure 5. In general, the system is divided into two subsystems, namely the feature extractor using the CNN baseline (marked with a black dotted line) and the modified classifier with a multi-task approach (marked with a blue dotted line).

We started our research by investigating several CNN baseline architectures for highly similar vehicle brand and model classifications. Next, we performed two stages for each selected baseline CNN architecture. Then, we compared the results obtained from the CNN baseline architecture and the proposed architecture to see the improvements brought by the proposed architecture. Initially, we trained the original VGG-16 using 2934 trainset images from the InaV-Dash dataset to classify the vehicle brand. At this stage, the training results show that the original VGG-16 was unable to reach convergence. Next, we conducted a second stage of training, still using the same architecture and trainset as before, but this time it was aimed at vehicle model classification. The results obtained at this stage are worse than before because the vehicle model classification task is more difficult than the vehicle brand classification task, especially in highly similar vehicle conditions. In this research, these two stages were also used on a few other original CNN architectures to obtain a baseline for the make and model domains.

In order to improve the performance across classification tasks, we implemented a proposed architecture to modify the original VGG-16. Changes were made by replacing the classifier part from the original VGG-16 with a classifier from the proposed architecture and leaving only five block convolutional layers from the original VGG-16 as a feature extractor part. After the feature maps of the last convolutional layer in the fifth block are obtained, these feature maps will be flattened using the global average pooling layer and mapped into vectors with a size of 1 × 512. After that, the feature vector will pass through two pairs of layers, namely a dense layer with 256 neurons and a dropout layer, to learn the common representation feature for all tasks. This feature will then be used as input for two different branches: the branch for classifying the vehicle model and the branch for classifying the vehicle make. The layers of these two branches are the same: there are two dense layers and two dropout layers. The dense layer in both branches has the characteristics of a fewer number of neurons. This concept is inspired by the convolutional layer block arrangement in VGG-16, where in each block the size of the feature map becomes smaller. This decreasing number of neurons is expected to be able to filter important features gradually without losing too much important information. The difference in the two branches lies in the number of neurons in the last layer, namely dense5, which functions as a classifier layer. The activation function used in dense5 is SoftMax, while in the other layers, the activation function of the linear unit rectifier is used. Since the classifier in the proposed architecture already employs a multitask approach, we use a joint loss function during training to calculate an error value from two task-specific branches. 

Modifications to the classifier part were also carried out for several original CNN architectures and were evaluated to see the improvements brought by the proposed architecture. This study uses several methods to evaluate the performance of the proposed method, such as accuracy scores, specificity, sensitivity, and the F1-Score, to provide a comprehensive performance of the proposed method. In the next section, each module used in the above proposed architecture will be explained in detail along with its parameter settings.

#### 4.3.1. Convolutional Layer

The convolution layer of the CNN plays a role in carrying out the feature extraction process for the given input by following Equation (1), where C is the convoluted matrix, I is the input image with dimensions m×n, and k is the kernel matrix of size i×j. In this research, the input image size is 224 × 224 and the kernel size is 3 × 3. The convolution operation is performed with stride = 1 and padding = “same” so that the output feature maps are the same size as the input feature maps.

(1) $$C\left(i,j\right)=\left(I\times k\right)\left(i,j\right)=\underset{m}{\sum }\underset{n}{\sum }I\left(m,n\right)K\left(i-m,j-n\right)$$

#### 4.3.2. Max-Pool Layer

The main objective of the max-pool layer is to reduce the size of the input while preserving its key features, and especially its texture feature [58]. Furthermore, because it is more similar to biological characteristics, it can alleviate the problems of over-fitting and vanishing gradient to some extent. The operations performed on the max-pool layer can be written as in Equation (2).

(2) $$y_{i}=max_{R\times R}\left\{y_{i}^{r\times r}\right\}f\left(r,r\right)\;$$

Based on Equation (2), the max-pooling operation result matrix $\left(y_{i}\right)$ is obtained from the max operation in the R × R region where $y_{i}^{r\times r}$ is the *i*-th output of a window measuring r×r and f(r,r) represents the window function of the setting blocks. In this research, the max-pool layer is placed at the end of each block convolutional layer except for the last block convolutional layer. The max-pool layer operation is executed with a window of size 2 × 2 and stride = 2 × 2 resulting in an output map that is twice as small as the input.

#### 4.3.3. Global Average Pooling Layer

Similar to the max-pool layer, the global average pooling layer reduces the dimensions of the input matrix without losing its key features, hence reducing the computational load. The difference is that global average pooling uses all the values in the matrix and takes the average as the final output. In this study, the global average pooling layer replaces the normal flattening process before features from the last convolutional layer (conv5_3) pass to the dense layer.

#### 4.3.4. Dense Layer

A dense layer is another name for a fully connected layer in the form of a 1-D array. In this study, the dense layer gets 1D input from the results of the global average pooling layer as described in the previous section. The number of dense layers and the chosen activation functions determine whether a network can solve non-linear problems [59]. This study uses eight dense layers in the classifier part of the proposed architecture, with two layers being outside the branch, three layers being inside the vehicle model recognition branch, and three more layers being inside the vehicle brand recognition branch. The number of nodes in each dense layer tends to shrink towards the output layer.

#### 4.3.5. Dropout Layer

The dropout layer was introduced by Hinton et al. [60] and is commonly used in conjunction with dense layers to overcome the over-fitting issue [61]. The dropout layer works by randomly dropping units and freezing their incoming and outgoing connections [62]. This can lower the computational cost and help find the most unique features of its inputs [63]. The selection process of units to be dropped is set using a probability of retention parameter p with a range values of p, 0<p<1. In this study, the p value used is p=0.1 for all the evaluated architectures.

#### 4.3.6. Activation Functions

An activation function is applied to an artificial neural network in order to help the network learn complex data patterns and solve non-linear problems [64]. In this study, two types of activation functions were used, namely the rectified linear unit (ReLU) and Softmax functions. The ReLU function is the most often used activation function [35], which returns a zero for an input on the origin and over the negative domain and returns a linear value on the positive domain [65], as can be seen in Equation (3).

(3) $$ReLU=\{\begin{array}{c} 0,x\leq 0\\ x,x>0 \end{array}$$

In the proposed architecture, the ReLU function is used on all convolutional layers and on several dense layers in each branch (dense1 to dense4). As for the output layer (dense5), the proposed architecture employs a Softmax activation function. The Softmax function is a combination of several sigmoid functions in the form of nodes [66], which can generate class probabilities for each task as output [67]. The probabilities are derived from the output of each sigmoid node, which can only yield values between 0 and 1. The node with the highest probability will determine the class that is used as the final network output. Mathematically, the Softmax function can be expressed as Equation (4):

(4) $$\sigma {\left(z\right)}_{j}=\frac{e^{z_{j}}}{\sum_{k=1}^{K}e^{z_{k}}}\;\mathrm{for}\;j=1,\ldots ,K$$

where $\sigma {\left(z\right)}_{j}$ is the probability value for the jth class, $e^{z_{j}}$ is the output of the jth node, $e^{z_{k}}$ is the output of each existing node, and K is the number of classes. In this study, the Softmax function is implemented in the last dense layer in each branch (dense5) with a value of *K* = 4 for the vehicle make recognition branch and *K* = 10 for the vehicle model recognition branch.

#### 4.3.7. Loss Function

The loss function is a function that is used to measure the success of the network in classifying during the training process. Since the proposed network can yield two outputs in one round of propagation, the loss function must also take this into account. Therefore, the author uses a combined loss function as in Equation (5):

(5) $$L_{joint}=\lambda_{1}\cdot L_{vehicle\_model\_loss}+\lambda_{2}\cdot L_{vehicle\_brand\_loss}\;$$

where $L_{joint}$ is the joint loss function for the proposed architecture, $L_{vehicle\_model\_loss}$ is the loss function produced by the vehicle model recognition branch, $L_{vehicle\_brand\_loss}$ is the loss function yielded by the vehicle make recognition branch, $\lambda_{1}$ and $\lambda_{2}$ are the two-branch loss weights. Meanwhile, $L_{vehicle\_model\_loss}$ and $L_{vehicle\_brand\_loss}$ are defined as the typical cross-entropy loss as in Equation (6):

(6) $$L=-log\left[P\left(y_{i}|x_{i},\theta \right)\right]\;$$

where $x_{i}$, $y_{i}$, and θ denote input, label, and the network parameter, respectively. The above loss function will be able to measure how well each branch in the proposed architecture works and give a better update to the weights so that convergence can happen more rapidly. 

### 4.4. Performance Evaluation Method

The performance evaluation of a machine learning classifier is a crucial aspect of any research because it shows how well the classifier can guess the input image correctly [68]. In this study, a categorical accuracy method was used to provide a single and general accuracy score for each task, i.e., vehicle make recognition and vehicle model recognition. The formula for calculating categorical accuracy scores in this study can be seen in Equation (7):

(7) $$Accuracy=\frac{Number\;of\;correct\;prediction}{Total\;number\;of\;prediction}$$

After knowing the classifier performance score in general, we conducted another evaluation to obtain the performance of the proposed method in guessing each class. This evaluation is conducted by creating a confusion matrix and calculating the values of true positive (TP), true negative (TN), false positive (FP), and false negative (FN). To make it easier to understand the meaning of the TP, TN, FP, and FN values used in this study, we will take as an example the calculation for the Toyota class. The TP value represents the number of images that are predicted to be Toyota and that are indeed from Toyota’s class ground truth. The TN value represents the number of images that are predicted to be a brand other than Toyota and have a non-Toyota ground truth. The FP value represents the number of incorrectly predicted images as belonging to the Toyota class, while the FN value represents the number of images in which the Toyota class image is incorrectly predicted as a class other than Toyota. After getting the TP, TN, FP, and FN values for all existing classes, the next step is to find the precision (PR) and recall (RE) values for each class using Equations (8) and (9).

(8) $$\mathrm{PR}=\frac{\mathrm{TP}}{\mathrm{TP}+\mathrm{FP}}$$

Precision (PR), also known as positive predictive value (PPV), denotes the frequency with which the model correctly predicts the class. It indicates the proportion of accurate model predictions.

(9) $$\mathrm{RE}=\frac{\mathrm{TP}}{\mathrm{TP}+\mathrm{FN}}$$

Recall (RE) describes the degree to which the positive predictions of a machine learning model correspond to reality. It measures the proportion of our ground truth classes that are also predicted by the model.

After the PR and RE values for each class are calculated, we used these values to calculate the F1-Score as a harmonic mean of precision and recall [69]. The F1-Score can help us demonstrate how the classification model is “confused” when making predictions [70]. The F1-Score value for each class ($F1Score_{i}$) can be calculated using Equation (10).

(10) $$F1Score_{i}=2\frac{\mathrm{PR}\times \mathrm{RE}}{\mathrm{PR}+\mathrm{RE}}$$

However, instead of using the F1-Score for each class as a classifier performance reference, this study will summarize the F1-Score values into two tasks, namely the F1-Score for the vehicle make recognition task and the F1-Score for the vehicle model recognition task. The technique used to summarize is the macro average technique. This technique is very appropriate to be applied to imbalanced datasets such as the InaV-Dash dataset, wherein the data are spread over several classes in an imbalanced manner but all classes have the same level of importance. The formula for calculating the F1-Score using the macro average technique in this study is shown in Equation (11).

(11) $$Macro\;Average\;F1Score=\frac{1}{n}\overset{n}{\underset{i=1}{\sum }}F1Score_{i}$$

where n is the number of classes in one task (i.e., n=4 for the vehicle brand recognition task and n=10 for the vehicle model recognition task) and i is the class in each task. 

## 5. Results and Discussion

### 5.1. Experimental Setup

We use the Python programming language, which runs on Windows 10, to conduct this research. The Keras Library, which is connected to the TensorFlow backbone, was utilized to construct all CNN architectures during the experiment. Although network weights can be acquired through the training of networks from scratch, in this study, pre-trained models were employed because the training from scratch is computationally complex and can be prohibitively expensive if it requires application-specific hardware. Moreover, the use of a pre-trained CNN with proper fine-tuning outperformed or, in the worst situation, performed comparably to a CNN built from scratch [71]. In this study, we used ImageNet pre-trained weight as a starting point for training on the proposed architectures. The initial value for the learning rate is set at 0.002 and the weight decay is set to 0.5. We trained the network for 50 epochs using the SGD optimizer with momentum = 0.9. The training procedure is conducted using a batch model with a batch size of 16. All of the results of the experiments were measured on a single computer with the specs shown in Table 2.

### 5.2. Experimental Results on Single-Task CNN

**Figure 6:**
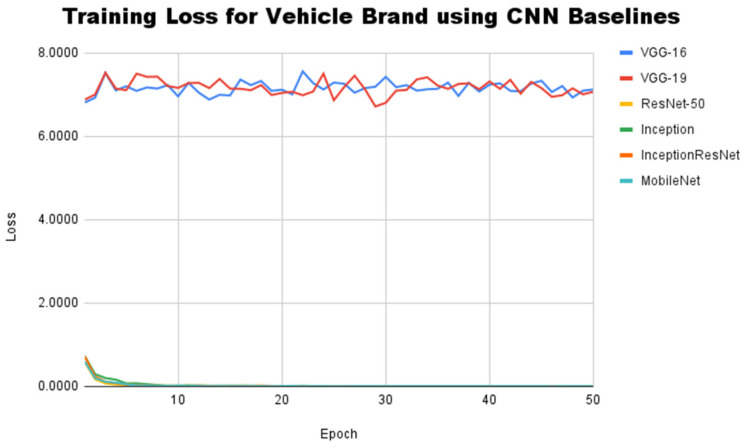
Training loss for vehicle brand classification using CNN baseline architectures.

This subsection will discuss the performance of the CNN baseline architecture in identifying the vehicle manufacturer and model in the InaV-Dash dataset. This subsection is divided into two experiments. The first experiment aims to see the performance of several CNN baseline architectures in the context of vehicle brand classification in the InaV-Dash dataset. In this experiment, each baseline architecture has two different versions of the output layer. The output layer of the first version is a SoftMax layer containing four neurons, which is used to identify the vehicle manufacturer, while the output layer of the second version is a SoftMax layer containing 10 neurons, which is used to identify the vehicle model. This experiment uses a learning curve as an evaluation measure for the CNN baseline architecture in the training process. Figure 6 shows the learning curve made by the training loss value for the task of recognizing the brand of a vehicle with the CNN baseline architecture.

According to Figure 6, the baseline CNN architecture of the ResNet, Inception, and MobileNet families is able to reach a convergent state after going through 16 epochs. However, the VGG-16 and VGG-19 architectures do not exhibit a trend towards convergence. VGG-16 and VGG-19 consistently produce high loss values during training, with significant differences compared to other architectures. After training VGG-16 and VGG-19 for vehicle brand classification, the lowest loss values are 6.92 and 7.20, respectively. This shows that the architecture of the VGG family is not able to find sufficient distinguishing features for each recognized vehicle manufacturer. To ensure the training results, the accuracy score and F1-Score were used as evaluation measures. This measurement was performed using a testset of the InaV-Dash dataset. A comparison of accuracy scores and F1-Scores for each CNN baseline architecture can be seen in Table 3. 

The accuracy score shows that there is a close relationship between the loss curve formed from the training results and the CNN’s ability to classify. For the vehicle brand classification task, four CNN baseline architectures that were able to achieve convergence on the learning curve, namely ResNet50, Inception, InceptionResNet, and MobileNet, were able to obtain an accuracy score above 93%. On the other hand, the VGG-16 and VGG-19 architectures that did not reach convergence on the learning curve only produced a 56% accuracy. However, when viewed in more detail, this 56% accuracy score is obtained by issuing the class prediction results as Toyota for all test images. This prediction result is clearly influenced by the imbalance condition in the InaV-Dash dataset, which has the highest number of images with the Toyota brand among other manufacturers. This can also be seen from the F1-Score for three classes other than Toyota, which obtained a score of 0.00, which means that VGG-16 and VGG-19 were not able to guess any image from the Daihatsu, Honda, and Suzuki classes correctly. 

ResNet-50 became the baseline architecture with the highest accuracy score, reaching 95.39%. The architecture of the Inception family, namely Inception and InceptionResNet, has a slightly lower accuracy than ResNet50, but it has an F1-Score of 1.00 for Honda manufacturers. This shows that the architecture of the Inception family is able to find unique characteristics for the vehicle image with the Honda manufacturer so that it can make predictions correctly. When viewed as a whole, the ResNet-50 and Inception-InceptionResNet architectures actually have the same ability to make predictions. Meanwhile, MobileNet has a slightly lower performance than ResNet and Inception. From the results of this evaluation, it is also known that the Daihatsu manufacturer is the most difficult class to predict. This could be influenced by the fact that vehicles from the Daihatsu manufacturer have many similarities with vehicles from the Toyota manufacturer when viewed from the point of view of the logo and body shape of the vehicle. This means some of the images of Daihatsu vehicles are wrongly classified as Toyota class.

**Figure 7:**
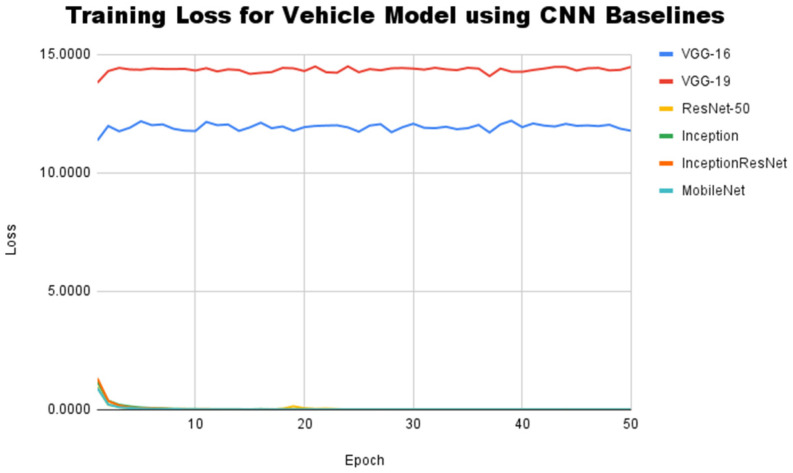
Training loss for vehicle model classification using CNN baseline architectures.

The next experiment is still aimed at looking at the performance of several CNN baseline architectures, but this time for the context of vehicle model classification on the InaV-Dash dataset. The learning curve generated by the CNN baseline architecture during training for vehicle model classification can be seen in Figure 7. 

The learning curve formed in the context of vehicle model classification is still in line with the learning curve generated in the context of vehicle brand classification. In this experiment, the four baseline CNN architectures, i.e., ResNet50, Inception, InceptionResNet, and MobileNet, were again able to achieve convergence in the training phase. Convergent conditions can be achieved by these four architectures after going through about 15 epochs of the training process. In contrast, VGG-16 and VGG-19 also exhibited poor results during training because they failed to reach convergence. The baseline architecture of the VGG family also produces a greater loss value than the previous experiment. The smallest loss values from VGG-16 and VGG-19 in training for the vehicle model classification task are 11.89 and 14.37, respectively. This is influenced by the vehicle model classification task, which is more complicated (10 classes) compared to the vehicle manufacturer classification task (4 classes). To find more complete evidence regarding the performance of the CNN baseline architecture, accuracy scores and F1-Scores were used again in this experiment. Table 4 shows the performance of each CNN baseline architecture used in the context of vehicle model classification in the InaV-Dash dataset testset.

Performance comparisons using accuracy scores still show the same result as the resulting learning curve. The VGG-16 architecture, which was not able to achieve convergence during training, was only able to produce an accuracy score of 25%. If viewed in more detail, this accuracy is obtained because the VGG-16 architecture tends to predict the test image as an Avanza class. Evidence of this can be seen from the F1-Score for the Avanza class, which reached 0.41. This phenomenon is also influenced by the imbalance condition in the InaV-Dash testset, wherein the image from the Avanza class dominates compared to other classes. Meanwhile, VGG-19 differs from VGG-16 in terms of predictive tendencies. VGG-19 tends to produce Brio-class predictions. This is reinforced by the accuracy of VGG-19, which only reaches 11%, or equivalent to the ratio of Brio class images on the InaV-Dash dataset testset. This trend can also be seen in the F1-Score belonging to the VGG-19 architecture, which is all zero except for the Brio class, which produces a value of 0.20.

On the other hand, the four architectures that managed to achieve convergence recorded a fairly good accuracy score, which is above 90% for the context of vehicle model recognition. ResNet50 again achieved the highest accuracy score of 95.95%, followed by Inception, which reached 93.88%. In this experiment, MobileNet was able to be slightly superior to InceptionResNet by a difference of about 1%. This shows that in the context of vehicle model recognition with a high degree of similarity, deeper layers in the architecture will not guarantee a better classification performance. Overall, the performance of these four architectures is not too different. This can be seen from the F1-Score values generated by these four architectures for each class of vehicle model, which are not too different. The results of this experiment also show that the Xenia vehicle model class is the most difficult class to identify correctly, with an average F1-Score of 0.79. This average is quite low compared to the average F1-Score for other classes, which is of at least 0.85. At the same time, the phenomenon strengthens the case that the high level of visual similarity for the vehicle models in the InaV-Dash dataset can cause network confusion and saturation. In this case, most of the images of the Xenia class, which failed to be predicted correctly, were misclassified as the Avanza class. In addition to the very similar visual appearance of the two classes, the imbalance condition also makes the network tend to provide more predictions of Avanza class compared to predictions of Xenia class. In the next section, we will discuss the performance of the proposed architecture in dealing with this problem. 

### 5.3. Experimental Results on Multi-Task CNN

**Figure 8:**
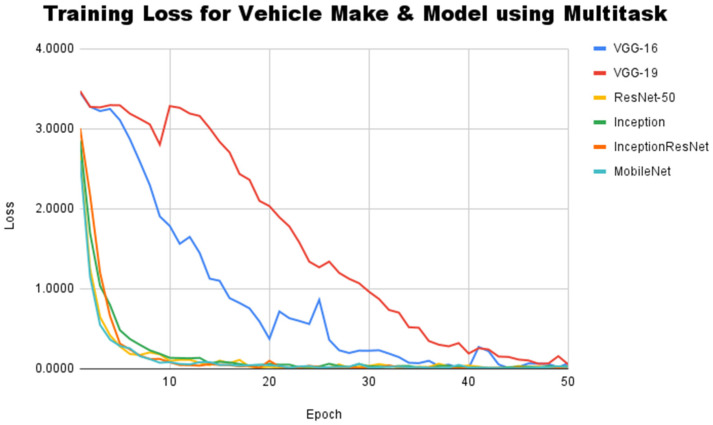
Training loss for both vehicle brand and vehicle model classification using proposed architectures.

This subsection describes the performance of the proposed multi-task CNN to recognize vehicle images taken from the InaV-Dash dataset testset. Since the proposed architecture already has a branch for each classification task to be worked on, the training process to identify vehicle brands and vehicle models can be carried out simultaneously. A learning curve is still a performance measurement technique for the proposed architecture during the training process. Since the proposed architecture already supports multi-task learning, the learning curve is drawn based on the combined loss values generated from the two branches. The learning curve for the proposed method can be seen in Figure 8.

In this experiment, the learning curve shows a different trend from the two previous experiments. Based on the learning curve that is formed, it is known that every existing line coincides with each other after going through 46 epochs, which indicates that all baseline architectures are able to reach convergence. This condition has never happened when using the single task learning approach in the two previous experiments. In other words, the proposed architecture is able to improve network performance during training, especially for networks that use the VGG-16 and VGG-19 architectures. The training results reveal that VGG-16 and VGG-19 achieved the lowest loss values of 0.05 and 0.32, respectively. This result is comparable to the loss levels achieved by four other architectures, which fall between 0.01 and 0.04. Even though the VGG family architecture does not have the lowest loss compared to other architectures, the best-performing architecture cannot be decided only by the loss value. We continue the evaluation by comparing each architecture’s accuracy scores to see how they compare in terms of performance. Table 5 shows a comparison of accuracy scores for vehicle model and brand recognition on the InaV-Dash dataset using the proposed architecture. To distinguish the baseline architecture from the proposed architecture, we add the MT code (short for Multi-Task) behind the CNN architecture name. Based on the accuracy score, the proposed architecture shows a very promising improvement in performance.

The training results are able to achieve convergence, as evidenced by an accuracy score of above 95% for vehicle brands and above 92% for vehicle models for all architectures. VGG-16, which in previous experiments always failed to achieve convergence in training, performed the best among other architectures with an accuracy score of 98.73% for vehicle brand recognition and 97.69% for vehicle model recognition. A significant increase also occurred in the VGG-19, which was able to produce an accuracy score of 96.58% for vehicle brand recognition. In the context of vehicle model recognition, VGG-19 is the architecture with the lowest score compared to other architectures. However, the application of the proposed method on VGG-19 provides the highest increase in accuracy, reaching 81.40%. The proposed method is also proven to be able to improve accuracy for the other four CNN architectures for both vehicle brand and vehicle model recognition tasks. Accuracy improvements for the other four CNN architectures are in the range of 0.5–5.9%. Moreover, F1-Score analysis can be used to see the recognition capabilities in more detail.

**Figure 9:**
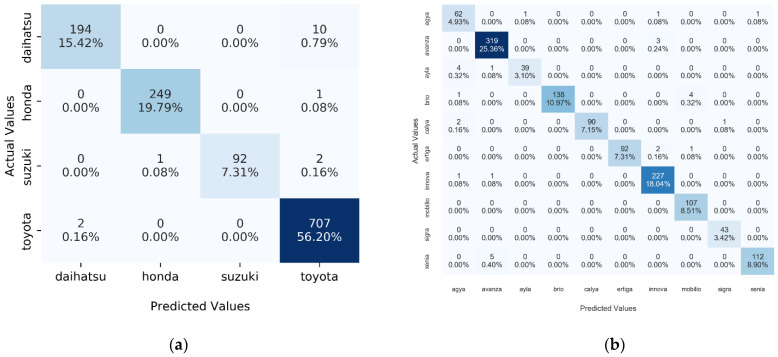
Confusion matrices generated based on VGG-16 MT results: (**a**) confusion matrix for vehicle brand classification task; (**b**) confusion matrix for vehicle model classification task.

After comparing the accuracy scores, we conducted a validity test on the architecture with the highest accuracy scores: VGG-16. A validity test was conducted to obtain specificity (PR) and sensitivity (RE) scores for each classification task. Initially, two confusion matrices are generated to facilitate the calculation process, as can be seen in Figure 9. After that, Equations (8) and (9) were used to calculate specificity and sensitivity scores for each class. Table 6 and Table 7 show the calculation results of the validity test for each classification task.

The results of the validity test show that the proposed method has a high level of specificity and sensitivity for the two tasks. All classes were able to achieve a classification score greater than 0.90 for the vehicle brand classification task. As for the vehicle model classification task, all classes were able to achieve specificity and sensitivity scores above 0.90 except for the Agya–Ayla vehicle model pair, which was at 0.89. This indicates that this vehicle model pair is the most challenging class to classify. Validity tests like this are also carried out for other CNN architectures. However, to provide a comprehensive view, we use the F1-score obtained from PR and RE scores. Table 8 compares the F1-Score for vehicle brand and model recognition on the InaV-Dash dataset using the proposed architecture. 

In the context of vehicle brand recognition, the VGG-16 architecture has the highest average F1-Score compared to other architectures. These results prove that the proposed method is able to handle the imbalance dataset problem, which in the previous experiment made the network always issue Toyota’s output as the dominant class in the testset. The VGG-16 is also able to correctly guess the entire image of the Honda manufacturer. The same improvement occurred in VGG-19, which now has an average F1-Score of 0.95 and can identify four car brand classes. The other four architectures also experienced an increase in F1-Score, although it was not as significant as the increase in the VGG architecture. The increase in F1-Score is most visible for the Daihatsu brand vehicle class, which is the most difficult vehicle brand class to identify. This indicates that the proposed method is able to recognize the image of the Daihatsu class better. Moreover, the proposed method does not reduce the network’s ability to recognize the Honda vehicle brand class that reaches an F1-Score of 1.0 on the Inception and InceptionResNet architectures. This result also makes Honda the most recognizable vehicle brand. Meanwhile, Daihatsu, as the most difficult class to identify, is only 0.06 adrift in the average F1-Score compared to Honda’s vehicle brand.

The pattern of performance improvement also occurs in the context of vehicle model recognition using the proposed method. With an average F1-Score of 0.97 for the vehicle model recognition task, the VGG-16 remains the architecture with the highest performance. This value also makes VGG-16 record the highest average F1-Score increase compared to the previous experiment, up to 0.93. The second highest F1-Score increase was recorded by VGG-19 with an increase of up to 0.88. As for the other four architectures, the increase occurred in the range of 0.01–0.08. Although the increase in F1-Score is less than 1%, the use of the proposed method is still able to improve performance for all existing vehicle model classes. This can be proven from the fact that the vehicle class of the Xenia model is no longer the hardest to identify. Xenia has a slightly higher average F1-Score than the Agya and Ayla, 0.92 compared to 0.91, which makes Agya–Ayla the hardest vehicle model to classify. Since Agya–Ayla is one of the dataset’s highly similar vehicle model pairs, this illustrates that highly similar vehicle models cause network difficulties. However, Agya–Ayla’s class average is not too bad as it is only 0.07 of the Brio–Mobilio and Innova, which have the highest averages.

Overall, the proposed method improves both tasks, especially on VGG-16 and VGG-19 architectures. The improvements occur because the proposed method is able to take the two architectures out of the local minima point and towards convergence. The multi-task learning method can also manage imbalances in the dataset that is being used.

## 6. Limitations

This study focuses on the vehicle make and vehicle model classification of highly similar vehicles using CNN-based architecture and a multi-task learning approach. Despite encouraging results, the proposed method failed to classify certain images due to certain conditions. Table 9 shows some examples of images that the proposed method did not correctly classify.

In Table 9, Correct Class represents the ground truth value, while Predicted Class represents the output from the proposed method. There are six sample images that failed to be classified by the proposed method. Samples 1 through 4 indicate that lighting conditions can considerably impact classification results. From these samples, we can see that the lighting conditions make the area around the manufacturer’s logo less clear, which leads to incorrect classification results. Sample 5 shows a classification failure because the logo emblem on the car has been modified, particularly in terms of its color. The use of this changed emblem is both a limitation of this research and a challenge for future research in this area. Sample 6 is an example of the most facelifted version of the Ayla car model, of which there are not many photos in the dataset. This condition causes the classifier to classify this image as Agya (its vehicle model pair) because Agya has many more photos in the dataset. We believe this problem can be solved by adding vehicle images with lighting condition cases and modified part cases, as well as balancing the number of images in the dataset for each existing class.

In practice, the proposed method is already rather good at recognizing vehicle images in real-world traffic scenes. This is because the proposed method uses images that come directly from the dashboard camera without any image enhancement processes. Based on the evaluation results, the method works very well in several CNN baseline architectures, which bodes well for its practical application. However, in this study, we have not built a computer that can be placed in a car and used as a processing unit to run our proposed method.

## 7. Conclusions

In this paper, a novel architecture with a multi-task learning approach has been proposed for a highly similar vehicle model. Utilizing the CNN baseline architecture as a feature extractor, a novel architecture is able to produce output for two tasks simultaneously, namely vehicle brand recognition and vehicle model recognition. VGG-16 MT is the proposed architecture with the best performance compared to the other five architectures. The VGG-16 MT reaches an accuracy score of 98.73% for the vehicle brand classification task and 97.69% for the vehicle model classification task. In terms of validity, the VGG-16 MT produces a macro average F1-Score of 0.98 and 0.97 for the vehicle brand and vehicle model classification tasks, respectively. In this study, it is known that Daihatsu is the most difficult brand to classify and Agya–Ayla is the most difficult model pair to classify. Overall, the proposed method has many advantages over the baseline CNN architecture. First, the proposed method improves the CNN’s basic architecture by preventing the network from becoming trapped in a local minimum while it is being trained. Second, the proposed method can deal with unbalanced and highly similar vehicle datasets. It is inevitable that the number of car models in the real world will never be balanced, and some of them look very similar. Last but not least, the proposed method uses a multi-task learning approach to provide a more efficient solution for vehicle brand and vehicle model classification. Multi-task learning allows for the proposed method to perform feature extraction once and then send the results to each branch to perform classification.

The following aspects are currently the subject of ongoing work: First, we will improve the InaV-Dash dataset by expanding the vehicle model and vehicle brand. Due to the large number of vehicle models and the fact that almost every year the manufacturer launches a facelifted version of the existing vehicle model, we are challenged to implement a database-updating procedure such as the CARUS system [3] at the dataset expansion stage. Although this procedure is derived from mineral classification problems, it can be applied to any classification problem. This procedure will determine whether a new image needs to be added into the dataset after the image is recognized by the classifier. If the new image’s features are significantly distinct from those of the existing images, it will be added to the dataset. This procedure should make it easy to add more data to a dataset and create a feature-rich image dataset. Second, we will create a system that combines the proposed method with online training and prediction capabilities using input in the form of a video stream from the dashboard camera. This improvement will bring the proposed method closer to its practical application.

## Figures and Tables

**Table 1:** InaV-Dash dataset in statistics.

Vehicle Make	Vehicle Model	Trainset	Testset
Toyota	Agya	143	65
Toyota	Calya	196	93
Toyota	Avanza	767	322
Toyota	Innova	526	229
Daihatsu	Ayla	138	44
Daihatsu	Sigra	117	43
Daihatsu	Xenia	277	117
Suzuki	Ertiga	235	95
Honda	Brio	323	143
Honda	Mobilio	212	107
		2934	1258

**Table 2:** Hardware and software.

Item	Content
Processor	Intel core i7-6700(CPU) with 3.4 GHz
Graphical Processing Unit (GPU)	NVIDIA GeForce GTX 980 Ti
Memory	8.0 GB
Operating System	Windows 10
Python	Python 3.6.4
Cuda	CUDA 9.0
CuDNN	CuDNN 7.0.5

**Table 3:** Performance comparison for vehicle brand classification using CNN baseline architectures.

No.	CNN Architecture	Vehicle Brand Accuracy	F1-Score Daihatsu	F1-Score Honda	F1-Score Suzuki	F1-Score Toyota	Macro Average F1-Score
1.	VGG-16	56.36	0.00	0.00	0.00	0.72	0.18
2.	VGG-19	56.36	0.00	0.00	0.00	0.72	0.18
3.	ResNet50	95.39	0.87	0.99	0.98	0.96	0.95
4.	Inception	94.83	0.85	1.00	0.98	0.96	0.95
5.	InceptionResNet	94.75	0.85	1.00	0.98	0.95	0.95
6.	MobileNet	91.49	0.75	0.99	0.98	0.93	0.91

**Table 4:** Performance comparison for vehicle brand classification using CNN baseline architectures.

No.	CNN Architecture	Vehicle Model Accuracy	F1-Score Agya	F1-Score Avanza	F1-Score Ayla	F1-Score Brio	F1-Score Calya	F1-Score Ertiga	F1-Score Innova	F1-Score Mobilio	F1-Score Sigra	F1-Score Xenia	Macro Average F1-Score
1.	VGG-16	25.60	0.00	0.41	0.00	0.00	0.00	0.00	0.00	0.00	0.00	0.00	0.04
2.	VGG-19	11.37	0.00	0.00	0.00	0.20	0.00	0.00	0.00	0.00	0.00	0.00	0.02
3.	ResNet50	95.95	0.93	0.96	0.89	0.97	0.97	0.98	0.98	0.99	0.94	0.91	0.95
4.	Inception	93.88	0.88	0.93	0.87	0.99	0.92	0.98	0.99	0.99	0.84	0.80	0.92
5.	InceptionResNet	90.70	0.80	0.90	0.80	0.97	0.91	0.97	0.97	0.95	0.81	0.75	0.88
6.	MobileNet	91.73	0.87	0.90	0.83	0.99	0.92	0.99	0.99	0.99	0.80	0.69	0.90

**Table 5:** Vehicle brand and vehicle model recognition accuracy scores were obtained from the proposed method.

No.	CNN Architecture	Vehicle Brand Accuracy	Vehicle Model Accuracy
1.	VGG-16 MT	98.73	97.69
2.	VGG-19 MT	96.58	92.77
3.	ResNet50 MT	97.62	96.50
4.	Inception MT	96.90	96.66
5.	InceptionResNet MT	97.38	96.66
6.	MobileNet MT	96.42	95.47

**Table 6:** Validity test result for vehicle brand classification task from VGG-16 MT.

No.	Vehicle Brand	Specificity/Precision (PR)	Sensitivity/Recall (RE)
1.	Daihatsu	0.99	0.95
2.	Honda	1.00	1.00
3.	Suzuki	1.00	0.97
4.	Toyota	0.98	1.00

**Table 7:** Validity test result for vehicle model classification task from VGG-16 MT.

No.	Vehicle Model	Specificity/Precision (PR)	Sensitivity/Recall (RE)
1.	Agya	0.89	0.95
2.	Avanza	0.98	0.99
3.	Ayla	0.97	0.89
4.	Brio	1.00	0.97
5.	Calya	1.00	0.97
6.	Ertiga	1.00	0.97
7.	Innova	0.97	0.99
8.	Mobilio	0.96	1.00
9.	Sigra	0.98	1.00
10.	Xenia	0.99	0.96

**Table 8:** The F1-Score results for vehicle brand and vehicle model recognition using the proposed method.

No.	CNN Architecture	F1-Score Vehicle Brand					F1-Score Vehicle Model										
		Daihatsu	Honda	Suzuki	Toyota	Macro Average	Agya	Avanza	Ayla	Brio	Calya	Ertiga	Innova	Mobilio	Sigra	Xenia	Macro Average
1.	VGG-16 MT	0.97	1.00	0.98	0.99	0.98	0.92	0.98	0.93	0.98	0.98	0.98	0.98	0.98	0.99	0.97	0.97
2.	VGG-19 MT	0.94	0.97	0.92	0.98	0.95	0.80	0.97	0.78	0.93	0.92	0.92	0.96	0.91	0.84	0.93	0.90
3.	ResNet50 MT	0.93	0.99	0.99	0.98	0.97	0.90	0.96	0.92	0.99	0.95	0.99	0.99	1.00	0.95	0.91	0.96
4.	Inception MT	0.91	1.00	0.99	0.97	0.97	0.96	0.96	0.94	0.99	0.97	0.99	0.99	0.99	0.96	0.88	0.96
5.	InceptionResNet MT	0.92	1.00	0.99	0.98	0.97	0.94	0.96	0.95	0.99	0.96	0.99	0.98	1.00	0.94	0.92	0.96
6.	MobileNet MT	0.90	0.99	0.96	0.97	0.96	0.92	0.95	0.92	0.99	0.96	0.96	0.98	0.98	0.93	0.88	0.95

**Table 9:** Sample images that are incorrectly classified by the proposed method.

**Sample Image**	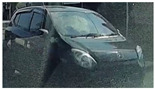	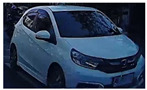	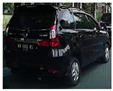
**Sample Name**	sample 1	sample 2	sample 3
**Correct Class**	Daihatsu Ayla	Honda Brio	Daihatsu Xenia
**Predicted Class**	Toyota Agya	Honda Mobilio	Toyota Avanza
**Sample Image**	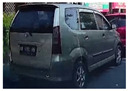	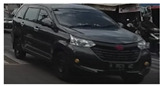	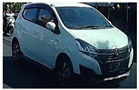
**Sample Name**	sample 4	sample 5	sample 6
**Correct Class**	Daihatsu Xenia	Daihatsu Xenia	Daihatsu Ayla
**Predicted Class**	Toyota Avanza	Toyota Avanza	Toyota Agya

## Data Availability

Not applicable.
